# Supplementary material for: Characterization of a Potent, Selective, and Safe Inhibitor, Ac15(Az8)2, in Reversing Multidrug Resistance Mediated by Breast Cancer Resistance Protein (BCRP/ABCG2)
Source: Int J Mol Sci. 2022 Oct 31;23(21):13261. doi: 10.3390/ijms232113261 (PMC9653733; doi:10.3390/ijms232113261)
Supplement: Supplementary file 1 [file ijms-23-13261-s001.zip › ijms-1953280-supplementary.pdf]

Supplementary Information

Characterization of a Potent, Selective, and Safe Inhibitor, Ac15(Az8)$_2$, in Reversing Multidrug Resistance Mediated by Breast Cancer Resistance Protein (BCRP/ABCG2)

Tsz Cheung Chong [1], Iris L. K. Wong [1], Jiahua Cui [1,2], Man Chun Law [1], Xuezhen Zhu [1], Xuesen Hu [1], Jason W. Y. Kan [1], Clare S. W. Yan [1], Tak Hang Chan [1,3,*] and Larry M. C. Chow [1,*]

1 Department of Applied Biology and Chemical Technology and State Key Laboratory of Chemical Biology and Drug Discovery, Hong Kong Polytechnic University, Hong Kong, China
2 School of Chemistry and Chemical Engineering, Shanghai Jiao Tong University, Shanghai 200240, China
3 Department of Chemistry, McGill University, Montreal, QC H3A 2K6, Canada
* Correspondence: tak-hang.chan@polyu.edu.hk (T.H.C.); larry.chow@polyu.edu.hk (L.M.C.C.); Tel.: +(852)-34008670 (T.H.C.); +(852)-34008662 (L.M.C.C.); Fax: +(852)-23649932 (T.H.C. & L.M.C.C.)

Table S1 Effect of **$Ac15(Az8)_2$** on body weight change of S1M180 bearing mice.

Groups	Treatments	Deaths	Weight change (%) on Day 55	Weight change (%) on Day 71
1	**$Ac15(Az8)_2$** solvent + Topotecan solvent	0/7[a]	37.4 ± 7.6	Not applicable
2	**$Ac15(Az8)_2$** sovlent + Topotecan (2mg/kg)	0/7[b]	17.2 ± 8.6	16.8 ± 9.1
3	**$Ac15(Az8)_2$** (45mg/kg) + Topotecan (2mg/kg)	0/7[b]	14.7 ± 5.8	7.9 ± 8.2

In each group, 7 mice were included and the weight change (%) was recorded on day 55 and 71. Weight change (%) value was presented as mean ± standard error of mean. On day 55, all mice in solvent treatment group (group 1) were sacrificed because of over-sized tumor. The weight change (%) on day 71 of group 1 was therefore labeled as not applicable. [a]Animal death was counted on day 55 for group 1. [b]Animal death was counted on day 71 for groups 2 and 3.

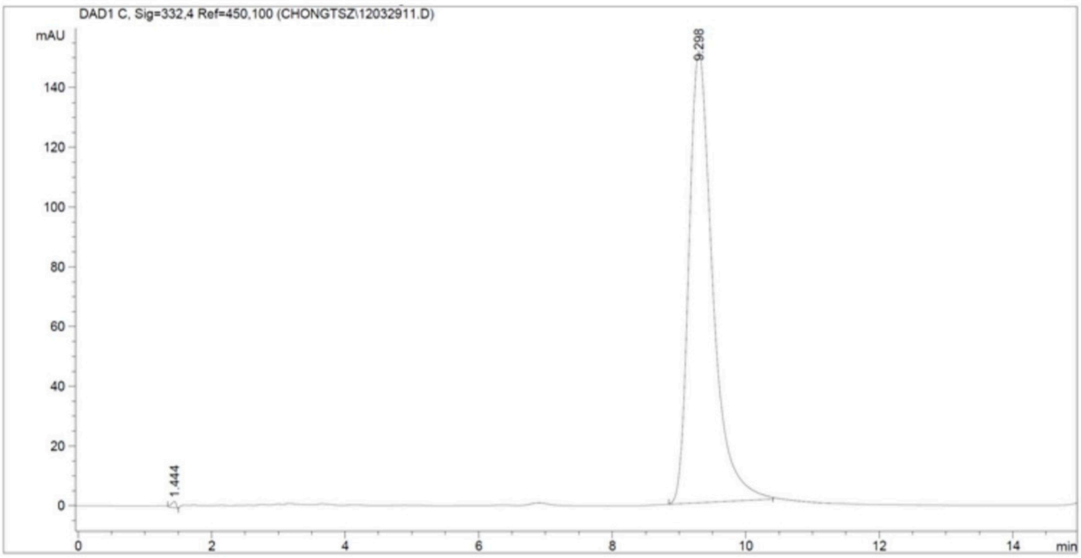

Figure S1 HPLC chromatogram of **Ac15(Az8)$_2$**. HPLC equipped with an Agilent Prep-Sil Scalar column (4.6 × 250 mm, 5-micron); flow rate: 1 mL/min; UV detection: 332 nm with reference at 450 nm; mobile phase: gradient elution of mixed solvents of dichloromethane and methanol from 10%/90% to 1%/99% in 20 minutes; retention time: 9.3 minutes; purity: 99%.

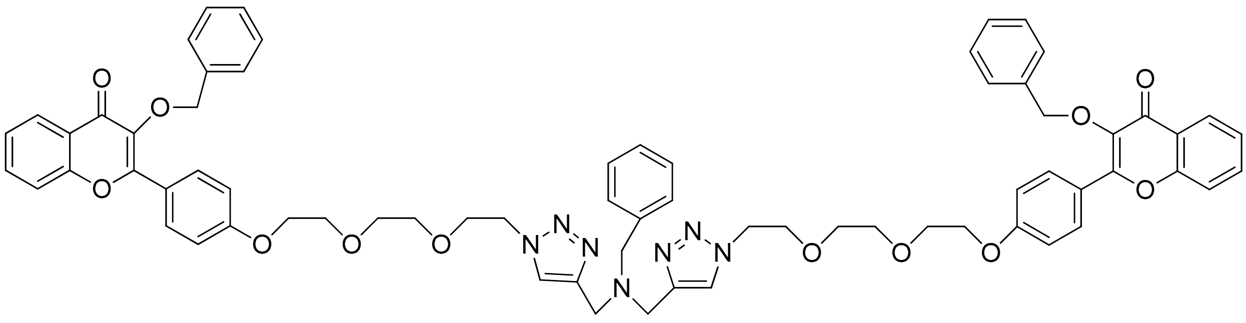

Ac15(Az5)$_2$

Figure S2 Chemical structure of **Ac15(Az5)$_2$**. It is used as an internal standard in UPLC-MS/MS.
